# Targeted Metabolic Therapy in Cancer: A Comprehensive Metabolic Treatment Strategy

**DOI:** 10.7759/cureus.108373

**Published:** 2026-05-06

**Authors:** Yahia Anane

**Affiliations:** 1 Metabolic Therapy and Cancer Metabolism, Metabolic Oncology Research LLC, Sheridan, USA

**Keywords:** cancer metabolism, cancer stem cells, fasting therapy, ketogenic diet, metabolic oncology, metabolic therapy, repurposed drugs, tumor hypoxia, tumor microenvironment

## Abstract

Cancer progression arises from the convergence of three domains: intrinsic metabolic and signaling rewiring within tumor cells, microenvironmental conditions that support survival and dissemination, and systemic metabolic dysfunction in the host. These layers collectively drive treatment resistance, immune evasion, and recurrence.

Targeted metabolic therapy (TMT) is proposed as a comprehensive therapeutic framework designed to simultaneously target all three domains of cancer progression. TMT targets key metabolic pathways implicated in tumor growth and survival such as glycolysis, glutaminolysis, IGF-1/PI3K-AKT-mTOR, angiogenesis, apoptosis resistance, and Wnt/β-catenin signaling, while also disrupting microenvironmental drivers including cancer stemness, metastasis, acidosis, hypoxia, and chronic inflammation. At the systemic (host) level, TMT corrects metabolic derangements - including hyperglycemia, systemic inflammation, and cachexia - that create a permissive environment for tumor progression.

The therapeutic model integrates multiple intervention categories. These include dietary and fasting strategies, repurposed pharmacological agents, nutraceuticals, and essential vitamins and minerals. Additional components encompass oxygen- and redox-modulating therapies and lifestyle optimization measures. This paper outlines the biological rationale for TMT and proposes a systems-level framework for applying coordinated metabolic pressure with the aim of improving treatment responsiveness, prolonging survival, and offering cancer patients a more durable path toward disease control and remission.

## Introduction and background

Cancer remains one of the leading causes of mortality worldwide, accounting for nearly 10 million deaths annually [1]. Despite major advances in molecular oncology and targeted therapies, long-term survival for many advanced solid and hematologic malignancies remains limited. A central reason is the remarkable plasticity of tumor cells: when one signaling or metabolic pathway is pharmacologically blocked, alternative pathways are rapidly engaged, enabling drug resistance and therapeutic escape [2]. This redundancy challenges any strategy that focuses on a single oncogenic target in isolation [2].

Over the past decades, converging evidence has reframed cancer not only as a genetic disorder but fundamentally as a metabolic disease. Warburg’s original observations highlighted the preference of tumor cells for aerobic glycolysis, even in the presence of adequate oxygen [3]. Subsequent work by Seyfried and others extended this concept, emphasizing the central role of dysregulated glucose and glutamine metabolism as core bioenergetic engines of malignancy [4]. More broadly, cancer cells integrate altered substrate utilization, redox control, mitochondrial reprogramming, and growth factor signaling into a coordinated metabolic network that sustains proliferation, invasion, and survival under stress [5].

Tumor behavior is further shaped by its microenvironment. Cancer stem cell compartments, metastatic programs, lactate-driven acidosis, hypoxia, stromal interactions, and chronic inflammation collectively determine immune accessibility, metastatic potential, and treatment resistance. These microenvironmental drivers operate alongside intrinsic metabolic pathways, forming a tightly coupled ecosystem rather than a purely cell-autonomous process [2,5].

At the same time, many patients present with systemic metabolic dysfunctions that create a supportive environment for cancer progression. Chronic inflammation, hyperglycemia, and abnormal blood counts have all been associated with poorer outcomes and increased cancer-related mortality [6,7]. These host-level abnormalities weaken immune surveillance, amplify pro-tumor signaling (e.g., via IGF-1/PI3K-AKT-mTOR), and facilitate cachexia and treatment intolerance [6,7].

In this context, we propose targeted metabolic therapy (TMT) as a comprehensive therapeutic framework that explicitly addresses both tumor-intrinsic mechanisms and host metabolic vulnerabilities. TMT rests on two interlocking pillars: blockade of dominant metabolic and signaling hubs (including glycolysis, glutaminolysis, angiogenesis/HIF signaling, IGF-1/PI3K-AKT-mTOR, Wnt/β-catenin, apoptosis resistance, and immune evasion, as well as cancer stemness, metastasis, acidity, hypoxia, and inflammation); and restoration of patient metabolic health by correcting systemic dysfunctions that fuel tumor growth.

To operationalize this concept, TMT integrates: dietary and fasting strategies (ketogenic diets, intermittent and prolonged fasting); repurposed drugs with defined metabolic and signaling effects (ivermectin, fenbendazole and mebendazole, itraconazole, metformin, low-dose naltrexone, baby aspirin); multi-target nutraceuticals (curcumin, berberine, thymoquinone, omega-3 fatty acids, modified citrus pectin, β-glucan-containing medicinal mushrooms, lactoferrin); vitamins and key minerals with documented anti-cancer or host-protective roles (vitamin D, zinc, selenium, magnesium); adjunct redox and oxygenation therapies (cannabidiol, sodium bicarbonate, hyperbaric oxygen); and lifestyle optimization (exercise, sleep regulation, structured stress management).

This paper synthesizes the mechanistic rationale and supporting evidence for TMT interventions within a unified metabolic framework. It presents a pathway-based schema for constructing rational, low-toxicity, multi-target therapeutic protocols. Interventions are aligned with specific tumor pathways, microenvironmental drivers, and host metabolic dysfunctions. Through this coordinated approach, TMT aims to restrict tumor adaptive escape mechanisms and promote durable disease control.

## Review

Cancer growth and signaling pathways

Cancer cells survive and expand by rewiring their metabolic and signaling pathways. These networks provide constant fuel, raw materials for new cells, and protection against death signals and immune attack. Unlike healthy cells, which rely on balanced energy production, tumors show extreme metabolic plasticity: if one growth route is blocked, another is activated [8-10]. This adaptability explains why conventional therapies often fail.

Among the many alterations described, a few stand out as the most universal drivers of cancer growth: glycolysis, glutaminolysis, angiogenesis, apoptosis resistance, and immune evasion. These core mechanisms represent the main targets of TMT. In the following subsections, we summarize the main metabolic and signaling pathways that serve as universal drivers of cancer growth.

Glycolysis and the Warburg Effect

One of the oldest and best-documented features of cancer is its abnormally high glucose consumption. Tumor cells may use 10-15 times more glucose than normal cells to sustain rapid growth and division [8]. In addition, many cancers display marked overexpression of insulin receptors, particularly the IR-A isoform, making them hypersensitive to insulin and IGF-1 signaling [8,9]. This ensures continuous glucose uptake, even when nutrient levels are low.

Rather than relying on efficient mitochondrial energy production, cancer cells preferentially convert glucose into lactate even in the presence of oxygen - the classic Warburg effect [3,9]. This shift is not just about energy: it supplies building blocks for DNA, proteins, and lipids while lactate acidifies the surrounding tissue, helping tumors invade and suppress immune cells [10,11].

High glycolytic activity is consistent across cancers, forming the basis of fluorodeoxyglucose (FDG)-positron emission tomography (PET) imaging, where glucose uptake lights up tumors throughout the body [12]. Clinical studies show that elevated glycolysis markers such as GLUT1 and LDH-A are strongly associated with aggressive disease and poor survival [13,14].

Glutaminolysis

Glutamine serves as a second major fuel for many tumors, a phenomenon often referred to as glutamine addiction [15]. After uptake, glutamine is converted to glutamate and then to α-ketoglutarate, replenishing tricarboxylic acid (TCA) cycle intermediates to sustain energy production and anabolism when glycolytic carbons are diverted to biosynthesis [16,17]. It also provides nitrogen and carbon for nucleotide and amino acid synthesis, directly supporting rapid cell division [18]. In addition, glutamine is a key precursor for glutathione and other antioxidant defenses, helping malignant cells tolerate oxidative and metabolic stress [19]. Glutaminase (GLS1), which catalyzes the first step of this pathway, is frequently upregulated in cancer, and its inhibition reduces proliferation and tumorigenicity in preclinical models [20].

Angiogenesis and Hypoxia Response

As tumors grow beyond the limits of simple diffusion, they require new blood vessels to supply oxygen and nutrients. This is achieved through angiogenesis, driven largely by hypoxia-induced stabilization of HIF-1α and the subsequent induction of pro-angiogenic genes such as VEGF [21,22]. HIF-1α also enhances glycolytic capacity by upregulating glucose transporters and glycolytic enzymes, linking hypoxia directly to metabolic reprogramming [23]. Overexpression of HIF-1α has been documented in many human cancers and their metastases and is associated with more aggressive disease and poorer outcomes [24]. Thus, angiogenesis and hypoxia signaling jointly create the vascular and metabolic climate that sustains tumor growth.

Apoptosis Resistance

Malignant cells must evade programmed cell death to survive and proliferate. Under normal conditions, apoptosis eliminates cells with excessive DNA damage or oncogenic stress, maintaining tissue homeostasis [25]. Tumors frequently tilt the balance of BCL-2 family proteins toward survival, with overexpression of anti-apoptotic members such as BCL-2 and BCL-XL and reduced function of pro-apoptotic regulators like BAX and PUMA [26-28]. By preventing mitochondrial outer membrane permeabilization and caspase activation, this imbalance allows cancer cells to withstand genotoxic damage and cytotoxic therapies. Apoptosis-resistant phenotypes are consistently linked with treatment failure and worse prognosis across a range of malignancies [26-28].

Immune Evasion

Although the immune system can detect and destroy transformed cells, clinically apparent tumors have escaped this surveillance. Immune evasion is now recognized as a core hallmark of cancer and is tightly coupled to metabolic rewiring [29]. Within the tumor microenvironment, cancer cells compete with T cells for glucose and key amino acids, limiting the substrates required for effective immune responses [30]. Lactate accumulation and associated acidosis further suppress cytotoxic T-cell activity and favor regulatory or exhausted phenotypes [31]. Many tumors also upregulate immune checkpoint ligands such as programmed death-ligand 1 (PD-L1), which inhibit T-cell function and promote immune escape [32]. Collectively, these mechanisms establish an immunosuppressive environment that permits ongoing tumor growth.

IGF-1/PI3K-AKT-mTOR Pathway

The insulin and IGF-1 axis converges on the PI3K-AKT-mTOR pathway, a central regulator of cell growth, metabolism, and survival [33]. Activation of PI3K and AKT enhances glucose uptake, stimulates anabolic metabolism, and provides strong anti-apoptotic signals [34,35]. Downstream, mTOR integrates nutrient and growth factor cues to drive protein and lipid synthesis while suppressing autophagy, enabling rapid expansion of malignant cell populations [34]. Dysregulation of this pathway is common in cancer and is frequently associated with aggressive clinical behavior and therapeutic resistance [33,35]. Systemic metabolic states such as obesity and hyperinsulinemia can further amplify IGF-1/PI3K-AKT-mTOR signaling, directly linking host metabolism to tumor progression [36].

Wnt/β-Catenin Pathway and Other Mechanisms

Another major signaling route in cancer is the Wnt/β-catenin pathway, which governs cell fate, proliferation, and stemness. In normal tissues, β-catenin is tightly regulated, but in tumors, mutations or aberrant Wnt activation stabilize β-catenin, allowing it to accumulate in the nucleus and drive transcription of oncogenic programs [37,38]. This promotes uncontrolled growth and supports the maintenance of cancer stem cells (CSCs), which are often more resistant to therapy [37,38]. Wnt/β-catenin signaling also contributes to immune exclusion; tumors with high β-catenin activity frequently lack T-cell infiltration and respond poorly to immunotherapy [39].

While glycolysis, glutaminolysis, angiogenesis, apoptosis resistance, immune evasion, IGF-1/PI3K-AKT-mTOR, and Wnt/β-catenin are dominant growth engines, cancer progression involves many additional pathways. These include Hedgehog and Notch signaling, the MAPK/ERK cascade, MYC-driven transcriptional reprogramming, NRF2-mediated antioxidant responses, and the loss of tumor suppressors such as p53 [2,5,29]. Together, these mechanisms underscore the redundancy and adaptability of tumor biology. TMT therefore focuses on applying pressure to the most universal and targetable hubs rather than any single isolated pathway.

Microenvironmental and cellular drivers of progression

While intrinsic metabolic and signaling pathways power cancer cell survival, tumor progression is equally shaped by the surrounding microenvironment. Factors such as stem-like subpopulations, metastatic competence, extracellular acidity, and chronic inflammation create conditions that protect malignant cells, enable dissemination, and fuel recurrence. These processes operate at the interface between tumor and host physiology, and they are major contributors to treatment resistance and long-term disease persistence.

Cancer Stem Cells

A subset of tumor cells, CSCs possess self-renewal, differentiation potential, and high tumor-initiating capacity [40]. CSCs exhibit enhanced DNA repair, increased drug efflux activity, and heightened resistance to oxidative and metabolic stress, making them markedly more resilient than bulk tumor cells [41]. These cells often survive conventional chemotherapy or radiotherapy and can repopulate tumors, contributing to recurrence [42]. Metabolically, CSCs display flexibility, shifting between glycolysis and oxidative phosphorylation depending on environmental conditions [43]. Clinically, high CSC signatures correlate with treatment resistance, metastatic spread, and poor overall outcomes across multiple cancer types [44].

Metastatic Cascade

Metastasis accounts for over 90% of cancer-related deaths. It is a multistep biological program encompassing local invasion, epithelial-to-mesenchymal transition (EMT), intravasation, survival in circulation, extravasation, and colonization of distant organs. [45]. These steps are supported by metabolic and microenvironmental adaptations: glycolysis and lactate promote EMT and invasion, angiogenesis provides vascular entry points, and platelets shield circulating tumor cells from immune detection [46]. Adhesion mediators such as galectin-3 facilitate binding to the endothelium and promote metastatic seeding [47]. Although most disseminated cells fail to survive, the few that do establish secondary tumors define advanced malignancy.

Acidity and Lactate Microenvironment

A defining feature of the tumor microenvironment is extracellular acidosis, primarily generated by high glycolytic activity and lactate export. Acidic conditions enhance invasion by activating matrix-degrading proteases and promoting EMT [48]. Lactate-rich microenvironments also impair immune surveillance: cytotoxic T cells and natural killer (NK) cells function poorly under low pH, while immunosuppressive cell types such as Tregs and myeloid-derived suppressor cells (MDSCs) are favored [31,49]. Acidosis additionally supports CSC maintenance and survival, linking metabolic reprogramming directly to stemness and recurrence [48,49]. Thus, tumor acidity is a dynamic driver of progression, not merely a metabolic byproduct.

Chronic Inflammation

Chronic inflammation is both a driver and consequence of tumor progression. Many cancers exhibit elevated inflammatory cytokines and systemic markers such as C-reactive protein (CRP), which correlate with reduced survival [6,50]. Inflammation promotes DNA damage, angiogenesis, immune suppression, and EMT, thereby accelerating malignant progression [51]. It also contributes to the formation of pre-metastatic niches by recruiting bone-marrow-derived cells and remodeling distant tissues, making them receptive to circulating tumor cells [52]. Persistent inflammation therefore sets the stage for metastatic spread and disease recurrence [50-52].

Tumor Hypoxia (Low Oxygen Microenvironment)

Tumor hypoxia arises when rapidly proliferating cancer cells outstrip local oxygen supply. Hypoxia stabilizes HIF-1α and HIF-2α, which regulate genes involved in glycolysis, angiogenesis, metastasis, and stemness [22,23]. Hypoxic regions develop severe metabolic stress that normal cells cannot tolerate, yet cancer cells adapt by increasing glycolytic flux and suppressing mitochondrial respiration [22,23]. Hypoxia also promotes immune evasion by reducing cytotoxic T-cell infiltration and increasing regulatory immune cell populations [31]. Clinically, hypoxic tumors are associated with aggressive behavior, therapeutic resistance, and poor prognosis [24].

Host metabolic dysfunctions

Cancer progression is strongly influenced by the metabolic condition of the host. Systemic abnormalities - including inflammation, hyperglycemia, hematologic imbalance, nutrient deficiencies, circadian disruption, and broader metabolic dysfunctions - create physiological conditions that promote tumor growth and impair immune surveillance [6,7,50,51]. Addressing these dysfunctions is therefore essential to any comprehensive TMT framework.

Systemic Inflammation

Chronic inflammation is closely associated with poorer outcomes in cancer patients. Elevated CRP and erythrocyte sedimentation rate (ESR) reflect systemic inflammatory activation and predict reduced survival. Inflammation - driven by poor diet, obesity, sedentary lifestyle, toxins, or chronic infections - promotes tumor growth through angiogenesis, cytokine signaling, immune suppression, and extracellular matrix remodeling [50,51].

Hyperglycemia and Insulin Resistance

Hyperglycemia and hyperinsulinemia establish a metabolic environment that accelerates cancer growth. High glucose directly fuels glycolysis-dependent tumors, while elevated insulin and IGF-1 activate the PI3K-AKT-mTOR signaling axis, enhancing proliferation and resistance to apoptosis [36]. Epidemiological evidence shows that diabetes and insulin resistance increase both cancer incidence and mortality across multiple malignancies [53].

Abnormal Blood Counts

Hematologic abnormalities are common in cancer and reflect systemic metabolic and inflammatory disruption. Anemia contributes to intratumoral hypoxia, enhancing HIF-1α activity and promoting angiogenesis and treatment resistance. Thrombocytosis facilitates metastatic dissemination through platelet-mediated shielding of circulating tumor cells and support of endothelial adhesion [54]. Lymphopenia signals impaired immune competence and is consistently associated with reduced survival across advanced malignancies [55]. These abnormalities serve not only as prognostic markers but also as functional drivers of tumor progression.

Nutrient Deficiencies

Micronutrient deficiencies frequently accompany cancer and negatively influence host metabolism and immune regulation. Vitamin D insufficiency is linked to higher cancer incidence and poorer outcomes due to its roles in cellular differentiation and immune modulation [56]. Deficits in magnesium and omega-3 fatty acids impair mitochondrial function and promote pro-inflammatory signaling that supports tumor growth [57]. Additional deficiencies in zinc, selenium, and B vitamins reduce antioxidant capacity and DNA repair [57]. Together, these disturbances weaken host resilience and create a metabolic environment conducive to malignant progression.

Circadian and Sleep Disruption

Disruption of circadian rhythms is increasingly recognized as a metabolic driver of cancer progression. Melatonin - an oncostatic hormone regulating mitochondrial function, immune activity, and oxidative balance - is often suppressed in individuals with poor sleep or irregular schedules [58]. Chronic sleep disturbance elevates cortisol and inflammatory markers, creating a physiological landscape that favors tumor survival.

Muscle Wasting/Cancer Cachexia

Cancer cachexia is a multifactorial metabolic syndrome characterized by ongoing loss of skeletal muscle (with or without fat loss) that cannot be fully reversed by conventional nutrition support. It arises from the same systemic inflammatory environment that drives tumor progression: elevated cytokines such as IL-6 and TNF-α, chronic activation of innate immunity, and persistent acute-phase responses [51]. These inflammatory networks promote proteolysis and lipolysis, impair anabolic signaling, and increase resting energy expenditure, leading to progressive weakness, treatment intolerance, and poorer survival. 

Other Metabolic Dysfunctions

Beyond the major metabolic disturbances described above, cancer patients commonly exhibit additional systemic dysfunctions - including hypertension, thyroid dysfunction, gastrointestinal disturbances, hepatic and renal impairment, and dyslipidemia - that may further compromise the host's metabolic stability and contribute to disease progression [51,53].

Intervention strategies

Effective cancer management requires interventions that simultaneously target tumor-intrinsic pathways, disrupt microenvironmental drivers of progression, and correct systemic metabolic dysfunctions in the host [2,5,8]. TMT integrates dietary strategies, promising repurposed drugs, nutraceuticals, and adjunct metabolic therapies to create coordinated, multi-pathway pressure against malignant growth. The following sections outline the major components of this therapeutic framework.

Dietary and Fasting Approaches

Ketogenic diet (KD): A ketogenic diet restricts carbohydrates and shifts cellular metabolism toward fatty acid oxidation and ketone production. This metabolic state lowers circulating glucose and insulin, reducing activation of insulin/IGF-1-PI3K-AKT-mTOR signaling, a central driver of cancer proliferation [59,60]. Preclinical studies demonstrate slowed tumor growth and improved response to therapy under ketogenic conditions due to reduced IGF-1, lower inflammation, and increased oxidative stress in cancer cells [60]. Early clinical data in glioblastoma and other cancers indicate feasibility and metabolic benefits, though large trials are still underway [61].

Intermittent fasting (IF): Intermittent fasting cycles feeding and fasting periods, promoting metabolic switching from glucose to fatty acids and ketones. Fasting lowers glucose, insulin, and IGF-1 levels while activating autophagy and cellular stress-adaptation pathways [62]. Experimental evidence shows that IF sensitizes tumors to chemotherapeutic and radiotherapeutic stress while protecting normal tissues through differential stress resistance [63]. Small clinical studies report reduced treatment-related toxicity and improved tolerability among patients practicing structured fasting protocols [64].

Prolonged fasting and fasting-mimicking diets: Prolonged fasting (typically 48-72 hours) induces a deeper metabolic shift than IF, with marked reductions in circulating glucose, insulin, and IGF-1 and a concomitant rise in ketone bodies. This state activates cellular stress-response pathways and autophagy while attenuating pro-growth signaling, creating conditions in which normal cells become more stress-resistant, whereas cancer cells remain vulnerable to damage [62,63]. In preclinical models, cycles of prolonged fasting confer differential stress resistance, protecting healthy tissues and enhancing the cytotoxicity of chemotherapy against tumors [63]. Complementary work shows that repeated fasting reduces IGF-1/PKA signaling and promotes hematopoietic stem-cell-based regeneration and reversal of chemotherapy-induced immunosuppression, supporting its potential as an adjunct to systemic cancer therapy [65].

Repurposed Drugs

Repurposed drugs form an important component of TMT by targeting key signaling and metabolic pathways that support tumor growth [2,5]. Many medications originally developed for parasitic, infectious, or metabolic diseases have demonstrated anticancer effects through mechanisms such as inhibition of angiogenesis, disruption of mitosis, modulation of immune responses, and suppression of oncogenic signaling [66-85]. The following agents illustrate how repurposed pharmacologic therapies can interfere with multiple drivers of cancer progression.

Ivermectin: Ivermectin is a macrocyclic lactone widely used as an antiparasitic agent, now recognized for its multi-target anticancer activity. It inhibits Wnt/β-catenin and YAP/Hippo signaling - key regulators of CSC maintenance - thereby reducing stemness and tumor-initiating capacity [66]. Ivermectin also triggers mitochondrial dysfunction and reactive oxygen species (ROS) accumulation, resulting in apoptosis and cell-cycle arrest [67]. Additional studies show selective cytotoxicity toward malignant hematologic cells via multi-pathway suppression [68]. Collectively, preclinical and early experimental evidence suggests that ivermectin may target CSC biology, proliferative metabolism, and components of the immunosuppressive microenvironment, though clinical validation through large-scale trials remains limited.

Benzimidazoles, fenbendazole, and mebendazole: Fenbendazole and mebendazole are benzimidazole anthelmintics traditionally used to treat parasitic infections. Their anticancer activity stems from strong binding to β-tubulin, inhibiting microtubule polymerization and disrupting mitosis [69,70]. This also impairs glucose uptake by interfering with microtubule-dependent GLUT trafficking, targeting tumor metabolic flexibility. Mebendazole further suppresses angiogenesis by downregulating VEGF signaling [70]. Case reports and observational studies document tumor regression or stabilization in glioblastoma, colon, and lung cancers [71]. Preclinical and observational evidence suggests these agents may simultaneously disrupt mitosis, glycolysis, and angiogenesis, though prospective clinical trials are needed to confirm these effects.

Itraconazole: Itraconazole is a triazole antifungal medication that demonstrates potent anticancer effects through vascular and developmental signaling pathways. It inhibits angiogenesis by suppressing VEGFR signaling and endothelial proliferation [72]. Additionally, itraconazole blocks Hedgehog pathway activation, a driver of tumor growth, stemness, and chemoresistance [73]. In clinical settings, itraconazole has shown disease stabilization in prostate and lung cancer patients when used as adjunct therapy [74]. Its dual targeting of angiogenesis and oncogenic signaling fits well within multi-pathway metabolic treatment frameworks.

Metformin: Metformin is an oral biguanide used globally as a first-line treatment for type 2 diabetes. Its anticancer efficacy arises from activation of AMPK, leading to downstream inhibition of the mTOR pathway and suppression of anabolic metabolism required for tumor proliferation [75]. Large epidemiological analyses show diabetic patients on metformin have reduced cancer incidence and improved survival relative to those on other glucose-lowering medications [76]. Preclinical work shows metformin decreases tumor growth, enhances responses to chemotherapy and radiotherapy, and improves metabolic resilience by lowering systemic insulin and IGF-1 signaling [75,76].

Low-dose naltrexone (LDN): Naltrexone is an opioid receptor antagonist traditionally prescribed for addiction disorders; at low doses (1-4.5 mg), it exhibits anticancer immunomodulatory effects [77]. LDN transiently blocks opioid receptors, producing a rebound increase in endogenous opioids and activating the OGF-OGFr axis, which slows tumor cell proliferation [77-79]. It also reduces TLR4/NF-κB-mediated inflammatory signaling, lowering cytokine production and supporting anti-tumor immune activity [78]. Preclinical studies and early clinical observations suggest reduced tumor growth and improved immune tone, though large-scale trials are limited [77,79,80].

Baby aspirin: Low-dose aspirin (75-100 mg/day) is widely used for cardiovascular prevention due to its antiplatelet activity via irreversible COX-1 inhibition. Its anticancer relevance stems from reducing platelet activation and preventing platelet cloaking of circulating tumor cells - a critical facilitator of metastatic spread [81,82]. Aspirin also suppresses COX-2-derived prostaglandin E2, lowering inflammation, angiogenesis, and immunosuppression [83]. Robust clinical evidence, particularly in colorectal cancer, demonstrates reductions in cancer incidence, recurrence, and mortality with long-term low-dose aspirin use [84,85].

Nutraceuticals

Nutraceutical compounds represent an important adjunctive component of TMT. These agents modulate key biological processes implicated in cancer progression, including inflammation, oxidative stress, tumor metabolism, and immune regulation [2,5]. In contrast to conventional pharmaceuticals, nutraceuticals exert multi-targeted biological effects with favorable safety profiles, supporting their use as adjuncts in comprehensive cancer management. The compounds reviewed in this section - curcumin, berberine, thymoquinone, omega-3 fatty acids, modified citrus pectin, medicinal mushrooms, and lactoferrin - have demonstrated mechanistic relevance across major pathways of cancer progression [86-99].

Curcumin: Curcumin, the principal polyphenol in Curcuma longa, exhibits broad anti-cancer activity by suppressing NF-κB signaling, downregulating pro-inflammatory cytokines, and modulating glycolytic enzymes such as LDH-A and GLUT1 [86]. It also induces apoptosis through ROS generation and mitochondrial pathways. Preclinical models show inhibition across multiple cancer types, and clinical studies demonstrate safety and potential synergistic effects when combined with chemotherapy or radiotherapy [87].

Berberine: Berberine, an isoquinoline alkaloid found in Berberis species, activates AMPK, resulting in downstream inhibition of the mTOR pathway and suppression of glycolysis [88]. It reduces circulating glucose and improves insulin sensitivity, indirectly lowering the fuels available for tumor metabolism. Berberine also induces apoptosis and exhibits anti-proliferative effects in preclinical colorectal, breast, and liver cancer models [89].

Black seed oil (thymoquinone): Thymoquinone, the major bioactive compound of Nigella sativa, promotes apoptosis by modulating BCL-2 family proteins and enhancing ROS-mediated cell death [90]. It also attenuates inflammatory signaling, including IL-6, TNF-α, and NF-κB activation [91]. Preclinical and in vitro studies demonstrate inhibition of tumor growth, reduced angiogenesis, and enhanced sensitivity to chemotherapy agents, positioning thymoquinone as a multi-mechanistic metabolic modulator.

Omega-3 fatty acids: Eicosapentaenoic acid (EPA) and docosahexaenoic acid (DHA) exhibit anti-inflammatory and anti-proliferative effects by suppressing COX-2 activity, reducing NF-κB activation, and altering membrane lipid composition [92]. Incorporation of omega-3 fatty acids into tumor cell membranes disrupts lipid rafts and enhances responsiveness to oxidative and metabolic stress. Observational studies associate higher omega-3 intake with reduced cancer progression and improved quality of life [93].

Modified citrus pectin (MCP): MCP is a low-molecular-weight, absorbable form of pectin that inhibits galectin-3, a carbohydrate-binding protein implicated in metastasis, immune evasion, and tumor-stromal adhesion [94]. Preclinical work demonstrates reduced metastatic spread, enhanced NK cell activity, and modulation of the tumor microenvironment. Early clinical studies suggest MCP may slow prostate-specific antigen (PSA) progression in prostate cancer and improve disease stability in colon cancer patients [95].

Medicinal mushrooms (β-glucans): Medicinal mushrooms such as Trametes versicolor and Lentinula edodes contain β-glucans that activate innate immunity through dendritic cells, macrophages, and NK cells [96,97]. These polysaccharides enhance cytokine production, promote anti-tumor immune responses, and improve tolerance to conventional therapy. Randomized controlled trials in gastric and breast cancer show improved survival and reduced recurrence when mushroom extracts are used as adjuncts [96,97].

Lactoferrin: Lactoferrin is an iron-binding glycoprotein that restricts iron availability to tumor cells, thereby suppressing proliferation and oxidative metabolism [98,99]. It also stimulates NK cell and macrophage activity while reducing inflammatory cytokines. Experimental studies demonstrate inhibition of tumor growth, reduced metastasis, and improved immune competence in various cancer models [99].

Vitamins and Key Minerals

Micronutrient deficiencies are common in cancer patients and contribute to impaired immunity, metabolic dysfunction, and reduced treatment tolerance. Several vitamins and minerals exert direct or indirect anti-cancer effects by supporting mitochondrial function, DNA repair, antioxidative defense, and immune surveillance. Restoring optimal levels is therefore a foundational component of TMT.

Vitamin D: Vitamin D has well-described anti-cancer effects through regulation of proliferation, differentiation, apoptosis, and immune modulation. Low serum 25(OH)D is associated with increased incidence and mortality of several cancers, including colorectal, breast, and prostate cancer [100]. Mechanistically, activation of the vitamin D receptor (VDR) downregulates pro-proliferative signaling, promotes cell-cycle arrest, and enhances anti-tumor immune responses, supporting its role as a key host-protective factor in cancer progression [101].

Zinc: Zinc is required for DNA repair enzymes, p53 function, and normal apoptosis. Deficiency leads to genomic instability, impaired antioxidant defense, and dysregulated immune surveillance, all of which favor carcinogenesis [102]. Experimental and clinical data suggest that adequate zinc status supports anti-tumor immunity - particularly T-cell-mediated responses - and may reduce the risk or progression of certain cancers by maintaining genomic integrity and limiting oxidative DNA damage [103].

Selenium: Selenium, via its role in glutathione peroxidases and other selenoproteins, modulates oxidative stress, redox signaling, and immune function. Prospective and interventional studies indicate that adequate or supplemented selenium intake is associated with reduced risk of several cancers (e.g., prostate, lung, colorectal), although results vary by population and baseline status [104]. Selenium’s anti-cancer actions include limitation of ROS-mediated DNA damage, modulation of apoptosis, and enhancement of immune responses against tumor cells.

Magnesium: Magnesium participates in ATP metabolism, DNA repair, and regulation of insulin sensitivity. Epidemiologic studies have reported inverse associations between dietary magnesium intake and risk of colorectal and pancreatic cancers, suggesting a protective role against tumor development in metabolically stressed states [105]. By stabilizing genomic structure, supporting normal cell-cycle control, and improving insulin sensitivity, magnesium helps counter several host-level metabolic conditions that drive tumor progression.

Adjunct and Redox Therapies

Several adjunct therapies influence the biochemical conditions of the tumor microenvironment, such as hypoxia, acidity, redox balance, and immune accessibility. By modifying these factors, they increase metabolic stress on cancer cells and enhance responsiveness to broader metabolic strategies. The following modalities illustrate key approaches to microenvironmental modulation.

Cannabidiol (CBD oil): Cannabidiol exhibits multi-targeted anticancer activity, including induction of apoptosis, disruption of mitochondrial respiration, and enhancement of ROS. Preclinical data demonstrate inhibition of AKT/mTOR signaling and suppression of metastatic behavior across several tumor models [106]. CBD also exerts anti-inflammatory and immunomodulatory effects that may further normalize the tumor microenvironment and support the efficacy of metabolic interventions [107].

Sodium bicarbonate (baking soda): Extracellular acidosis contributes to invasion, immune evasion, and metastatic progression. Sodium bicarbonate buffers tumor acidity, increasing pH and reducing metastatic spread in preclinical models [108]. By neutralizing lactate-driven acidification, bicarbonate improves T-cell activation and reduces protease-mediated matrix degradation, thereby limiting invasion and enhancing microenvironmental compatibility for immune surveillance [48,108].

Hyperbaric oxygen therapy (HBOT): HBOT increases tissue oxygenation, counteracting tumor hypoxia - a major driver of glycolysis, angiogenesis, and treatment resistance. By elevating oxygen tension, HBOT reduces HIF-1α activity, enhances ROS-mediated vulnerability of cancer cells, and improves perfusion in metabolically stressed tissues [109]. Preclinical studies demonstrate slowed tumor progression and increased therapeutic responsiveness when HBOT is combined with metabolic strategies [109].

Lifestyle Optimization

Lifestyle modification plays a critical role in restoring host metabolic balance and enhancing treatment responsiveness. Exercise, sleep regulation, and stress management modulate systemic inflammation, glucose metabolism, immune competence, and neuroendocrine signaling - factors strongly linked to cancer progression. Integrating these practices into metabolic therapy improves both physiological resilience and clinical outcomes.

Exercise as metabolic therapy: Regular exercise improves insulin sensitivity, lowers fasting glucose, reduces chronic inflammation, and enhances mitochondrial function in muscle and immune cells [110]. These effects counteract several host metabolic dysfunctions associated with tumor progression. Observational studies consistently report reduced recurrence and improved survival among physically active patients, particularly in breast and colorectal cancers [111].

Sleep optimization: Sleep maintains circadian rhythm integrity and supports secretion of melatonin, a hormone with documented oncostatic effects. Disrupted sleep elevates cortisol and inflammatory cytokines, impairing immune function and creating a pro-tumor environment. Experimental and clinical evidence indicates that restoring healthy sleep patterns improves treatment tolerance and may slow tumor progression [112].

Stress management: Chronic psychological stress activates sympathetic signaling and elevates cortisol and catecholamines, promoting angiogenesis, immune suppression, and metabolic dysfunction. Stress also amplifies systemic inflammation, supporting tumor progression. Interventions such as meditation, mindfulness, and breathing practices reduce stress hormones, enhance parasympathetic tone, and improve immune balance. Studies suggest these approaches may indirectly influence tumor behavior through reductions in inflammatory and neuroendocrine signaling [113].

Putting it all together: TMT

Cancer progression emerges from the interaction of metabolic rewiring, microenvironmental pressures, and systemic host dysfunction. TMT integrates dietary strategies, repurposed drugs, nutraceuticals, vitamins, and adjunct metabolic therapies to apply simultaneous pressure across these pathways. Table 1 summarizes the dominant growth and signaling pathways targeted by TMT, together with their roles in cancer biology and their corresponding key interventions. Table 2 maps the major microenvironmental drivers of tumor progression to the TMT components designed to disrupt them. Table 3 outlines the host metabolic dysfunctions that create a permissive environment for cancer growth and specifies the interventions used to correct each one.

**Table 1:** Growth and Signaling Pathways Targeted by TMT Abbreviations: TMT: Targeted Metabolic Therapy; HIF-1α: Hypoxia-Inducible Factor 1-alpha; IGF-1: Insulin-like Growth Factor 1; PI3K: Phosphoinositide 3-Kinase; AKT: Protein Kinase B; mTOR: Mechanistic Target of Rapamycin; PD-L1: Programmed Death-Ligand 1; HBOT: Hyperbaric Oxygen Therapy; CBD: Cannabidiol. Table created by the authors.

Pathway / Mechanism	Role in Cancer	Key Interventions
Glycolysis (Warburg effect)	High glucose uptake, lactate accumulation, immune suppression	Ketogenic diet, intermittent fasting, prolonged fasting, fenbendazole, metformin, berberine, curcumin
Glutaminolysis	Alternative fuel source, nucleotide synthesis, redox control	Prolonged fasting, fenbendazole, mebendazole, curcumin, berberine
Angiogenesis / HIF-1α	Neovascularization, hypoxia adaptation, metabolic reprogramming	Itraconazole, curcumin, black seed oil, CBD oil, aspirin, HBOT
Apoptosis Resistance	Mitochondrial protection, evasion of programmed cell death	Ivermectin, fenbendazole, mebendazole, curcumin, black seed oil, selenium, CBD oil
Immune Evasion	PD-L1 signaling, nutrient competition, lactate-mediated suppression	Ivermectin, low-dose naltrexone, medicinal mushrooms, vitamin D, omega-3, lactoferrin
IGF-1 / PI3K–AKT–mTOR	Growth factor signaling, proliferation, metabolic activation	Ketogenic diet, fasting, metformin, berberine, magnesium, CBD oil
Wnt / β-Catenin Signaling	Stemness, therapy resistance, immune exclusion	Ivermectin, itraconazole, curcumin, berberine, black seed oil

**Table 2:** Microenvironmental Drivers Targeted by TMT Abbreviations: TMT: Targeted Metabolic Therapy; EMT: Epithelial-to-Mesenchymal Transition; ECM: Extracellular Matrix; CSC: Cancer Stem Cell; HIF-1/HIF-2: Hypoxia-Inducible Factor 1/2; HBOT: Hyperbaric Oxygen Therapy; LDN: Low-Dose Naltrexone. Table created by the authors.

Driver	Role in Progression	Key Interventions
Cancer Stem Cells	Tumor initiation, recurrence, therapy resistance, metabolic plasticity	Ivermectin, itraconazole, fenbendazole, mebendazole, curcumin, berberine, black seed oil, fasting, metformin
Metastasis	EMT, intravasation, platelet cloaking, endothelial adhesion, colonization of distant organs	Modified citrus pectin, ivermectin, baby aspirin, omega-3, curcumin, berberine, lactoferrin
Tumor Acidity (Lactate Microenvironment)	Immune suppression, invasion, ECM degradation, CSC maintenance	Ketogenic diet, fasting, sodium bicarbonate, omega-3, curcumin, HBOT
Chronic Inflammation	Cytokine signaling, angiogenesis, DNA damage, formation of pre-metastatic niche	Omega-3, curcumin, berberine, black seed oil, CBD oil, vitamin D, low-dose naltrexone
Tumor Hypoxia (Low Oxygen Environment)	HIF-1/HIF-2 activation, angiogenesis, immune exclusion, metastasis, therapy resistance	HBOT, itraconazole, ketogenic diet, fasting

**Table 3:** Host Metabolic Dysfunctions Targeted by TMT Abbreviations: TMT: Targeted Metabolic Therapy; CRP: C-Reactive Protein; ESR: Erythrocyte Sedimentation Rate; IGF-1: Insulin-like Growth Factor 1; PI3K: Phosphoinositide 3-Kinase; AKT: Protein Kinase B; mTOR: Mechanistic Target of Rapamycin; LDN: Low-Dose Naltrexone; Vit D: Vitamin D; Mg: Magnesium; Zn: Zinc; Se: Selenium. Table created by the authors.

Dysfunction	Effect on Cancer	Key Interventions
Systemic Inflammation (CRP, ESR)	Drives cytokine release, angiogenesis, DNA damage, immune suppression	Omega-3, curcumin, berberine, black seed oil, CBD oil, vitamin D, low-dose naltrexone
Hyperglycemia / Insulin Resistance	Feeds glycolytic tumors, activates IGF-1/PI3K–AKT–mTOR growth signaling	Ketogenic diet, intermittent fasting, prolonged fasting, berberine, metformin, magnesium, exercise
Abnormal Blood Counts (anemia, thrombocytosis, lymphopenia)	Hypoxia, metastasis promotion via platelets, impaired immunity	Ketogenic diet (iron-rich food), fasting-induced regeneration, omega-3, baby aspirin, vitamin D, selenium, zinc, lactoferrin, low-dose naltrexone
Nutrient Deficiencies (Vit D, Mg, Omega-3, Zn, Se)	Weakens immune function, antioxidant defense, DNA repair, metabolic balance	Vitamin D, magnesium, omega-3, zinc, selenium.
Circadian Disruption	Low melatonin, high cortisol, increased inflammation and metabolic stress	Sleep optimization, fasting alignment with circadian rhythm, vitamin D, stress reduction practices, magnesium
Muscle Wasting / Cancer Cachexia	Loss of muscle mass, mitochondrial decline, metabolic inefficiency, reduced immunity, poor survival	Exercise, omega-3, lactoferrin, black seed oil, ketogenic diet (protein sufficient)

In clinical practice, TMT protocols are not designed to deploy all listed interventions simultaneously. Rather, the framework serves as a structured reference from which individualized, multi-target combinations can be constructed. TMT can be applied alongside conventional oncological treatments - including chemotherapy, radiotherapy, immunotherapy, and surgery. The selection process begins with identifying the dominant pathways and microenvironmental drivers in a given patient, then prioritizing interventions that address multiple targets concurrently. Prioritizing such multi-mechanistic interventions maximizes therapeutic pressure while minimizing protocol complexity and potential toxicity.

Beyond mechanistic considerations, practical factors play a critical role in intervention selection. These include the cost and availability of individual compounds, the patient's comorbidity profile and organ function, and the treating physician's familiarity and clinical experience with specific interventions. Regulatory status, potential drug interactions with ongoing conventional treatments, and patient compliance must also be carefully evaluated. TMT is therefore best applied under the guidance of a clinician with expertise in metabolic oncology, who can adapt the framework to the individual patient's biological, clinical, and logistical circumstances, and monitor response through serial metabolic and laboratory assessments.

Limitations

The present review has several limitations that should be considered when interpreting the proposed framework. The majority of evidence supporting the individual components of TMT derives from preclinical studies, including in vitro cell culture experiments and in vivo animal models, which may not fully recapitulate human tumor biology or clinical outcomes. Clinical evidence for many of the proposed interventions remains limited to small pilot studies, observational data, or case reports, and large-scale randomized controlled trials are lacking for most agents discussed. The heterogeneity of cancer types, stages, and patient populations further limits the generalizability of the available data.

The safety profiles, optimal dosing ranges, and potential interactions between the multiple agents comprising a TMT protocol have not been systematically evaluated in clinical settings. The combined use of repurposed drugs, nutraceuticals, and dietary interventions alongside conventional oncological treatments requires careful monitoring and individualized clinical judgment. Furthermore, as a narrative review, the literature was selected based on clinical and scientific relevance, which may introduce some degree of selection bias in the evidence presented.

The TMT framework is therefore a conceptual and mechanistic model that requires prospective clinical validation before definitive conclusions regarding efficacy and safety can be drawn. Future research should prioritize well-designed clinical trials evaluating coordinated metabolic intervention protocols as adjuncts to standard oncological care.

## Conclusions

Cancer progression arises from the convergence of multiple interconnected mechanisms, including tumor-intrinsic metabolic rewiring, microenvironmental adaptation, and systemic host-level dysfunction, rather than from a single mutation or isolated pathway. The framework outlined in this paper repositions cancer as a systemic metabolic disease: tumors co-opt glycolysis, glutaminolysis, angiogenesis, survival signaling, and immune evasion while simultaneously exploiting acidic, hypoxic, inflammatory niches and a metabolically compromised host. Any strategy that focuses on one axis in isolation is therefore inherently vulnerable to redundancy and escape.

TMT is proposed as an integrative, systems-level response to this problem. By design, TMT does not rely on a single "magic bullet," but applies coordinated pressure across multiple levels: dietary and fasting strategies that restrict key fuels and growth signals; repurposed drugs that interrupt oncogenic and stemness pathways; nutraceuticals and micronutrients that modulate inflammation, redox balance, and immune competence; adjunct redox and oxygenation therapies that reshape the tumor microenvironment; and lifestyle interventions that restore circadian integrity, muscle mass, and global metabolic health. TMT can be applied alongside conventional oncological treatments - including chemotherapy, radiotherapy, immunotherapy, and surgery - offering an additional layer of metabolic pressure that complements rather than competes with standard care. This conceptual model serves as a practical tool for constructing treatment plans built around clear mechanistic objectives, linking specific interventions to defined tumor pathways, microenvironmental drivers, and host metabolic dysfunctions.

Clinically, this approach provides a blueprint for constructing rational, multi-target protocols rather than ad hoc combinations of "metabolic" tools. It allows treatment plans to be built around clear objectives - such as suppressing glycolysis, depleting glutamine dependence, normalizing lactate and pH, dampening inflammatory signaling, reversing cachexia, and reactivating immune surveillance - and then selecting overlapping interventions that converge on those targets. Because many components of TMT (dietary shifts, fasting schedules, repurposed agents, nutraceuticals, and lifestyle changes) can be monitored via routine blood markers, imaging, and functional assessments, the framework is inherently suited to iterative adjustment and longitudinal tracking.

At the research level, TMT offers a structured paradigm for designing prospective studies that evaluate metabolic interventions not as isolated adjuncts, but as coordinated programs aimed at defined metabolic and microenvironmental endpoints. At the patient level, it supports the development of personalized protocols that are mechanistically grounded, quantifiable, and adaptable over time. It must be acknowledged that the majority of evidence supporting TMT components currently derives from preclinical studies and early clinical observations, and that large-scale randomized controlled trials validating coordinated metabolic protocols remain limited. While further clinical validation is required, the evidence summarized here suggests that systematically targeting cancer metabolism, the tumor microenvironment, and host physiology in parallel has the potential to improve outcomes, reduce recurrence, and shift the trajectory of disease from uncontrolled progression toward durable control and, in select cases, complete remission.
